# The impact of education/training on nurses caring for patients with stroke: a scoping review

**DOI:** 10.1186/s12912-024-01754-x

**Published:** 2024-02-02

**Authors:** Yanjie Zhao, Yuezhen Xu, Dongfei Ma, Shuyan Fang, Shengze Zhi, Meng He, Xiangning Zhu, Yueyang Dong, DongPo Song, Atigu Yiming, Jiao Sun

**Affiliations:** 1 School of Nursing, Jilin University, No.965 Xinjiang Street, Changchun, 130021 Jilin People’s Republic of China; 2 School of Nursing, Xinjiang Medical University, No.567 Shangde North Road, Urumqi, 830000 Xinjiang People’s Republic of China

**Keywords:** Education, Training, Professional competence, Nurses, Stroke, Scoping review

## Abstract

**Background:**

Stroke survivors have complex needs that necessitate the expertise and skill of well-trained healthcare professionals to provide effective rehabilitation and long-term support. Limited knowledge exists regarding the availability of specialized education and training programs specifically designed for nurses caring for stroke patients.

**Aim:**

This review aims to assess the content and methods of training for nurses caring for stroke patients, examine its impact on both nurses and patients, and identify key facilitators and barriers to its implementation.

**Methods:**

We conducted a comprehensive scoping review by reviewing multiple databases, including PubMed, Cumulative Index to Nursing and Allied Health Literature, PsycINFO, Embase, Web of Science, Scopus, ProQuest Dissertations and Theses, Google Scholar, and Cochrane databases. Data extraction and narrative synthesis were performed following the Preferred Reporting Items for Systematic Reviews and Meta-Analyses Extension for Scoping Reviews guidelines.

**Results:**

Seventeen articles were included in this review. We found that education/training not only enhanced patients' self-care abilities, nursing outcomes, and satisfaction, but also had a positive impact on the knowledge, skills, and practices of nurses. The obstacles to education/training included feasibility and cost-effectiveness, while the driving factors were management support and participation, professional education/training, and controlled environment creation.

**Conclusions:**

This review highlights the crucial role of education/training in enhancing stroke care provided by nurses. Effective education/training integrates various educational methods and management support to overcome implementation barriers and optimize clinical practice benefits. These findings indicate the necessity of universal and consistent stroke education/training for nurses to further improve patient outcomes in stroke care.

**Supplementary Information:**

The online version contains supplementary material available at 10.1186/s12912-024-01754-x.

## Introduction

Stroke is a primary cause of mortality and disability worldwide, and prompt medical management by specialists can improve the cure rate and minimize the disability, mortality, and recurrence rates [1]. The provision of care in stroke units is widely recognized for its positive impact on the prognosis of stroke patients [2]. This can be attributed to the presence of multidisciplinary teams equipped with extensive expertise, skills, and experience in stroke management. According to the Guidelines for Stroke Management, healthcare professionals involved in stroke care should possess a strong sense of service, expertise, and effective communication skills. Additionally, professional education and training should be offered to staff lacking the necessary knowledge or competencies [3]. Nurses play a crucial role as stakeholders and team members in the comprehensive stroke care system, influencing all aspects of care, from initial assessments and symptom recognition to treatment, rehabilitation exercises, early warning monitoring, psychological support, and end-of-life care [4, 5]. Consequently, stroke nursing staff require comprehensive training and education to ensure their capacity to deliver high-quality care to stroke patients. The development and implementation of stroke education and training programs for nurses are paramount in achieving high-quality stroke care and fostering positive patient prognosis [6].

Currently, the limited number of nurses worldwide who have received stroke education and training poses challenges in delivering high-quality care for individuals with stroke. In response to this issue, several developed countries, such as the UK with its National Stroke Strategy, have recognized the importance of nationally recognized, quality-assured, and replicable stroke education and training programs for healthcare professionals to ensure their proficiency and expertise [7]. Consequently, a Stroke-Specific Education Framework was established [8]. In developing countries like China, where stroke has a high prevalence, effective prevention and treatment of stroke have become crucial objectives for the overall population health. Consequently, in line with the decision-making and implementation of initiatives like the "Healthy China 2030 Plan" and "Action Plan for Implementing Healthy China," it is recommended that large healthcare institutions establish a "Brain Heart Health Manager Training Course" instructed by stroke experts [9]. This training should cover a comprehensive range of stroke management approaches at different stages. These strategies serve as valuable benchmarks and training guidelines for the development of stroke education programs for nurses.

The education and training of stroke caregivers pose significant challenges. These challenges arise due to the constantly changing conditions of individuals with stroke, as well as the presence of physical dysfunction, cognitive impairment, and psychosocial problems [10, 11]. Notably, cognitive impairment can be difficult to detect in the early stages [12]. Current education and training for nurses who care for stroke patients primarily consist of in-service training and continuing education courses [13]. However, there are notable issues with this approach, including limited educational resources such as inadequate materials, training programs, and support systems. Additionally, there is a need for the integration of existing educational methods to maximize their effectiveness and equip nurses with the necessary knowledge and skills for effectively managing stroke patients [14]. Research suggests that the effectiveness of education and training for stroke nursing staff can be enhanced through evidence-based guidelines, interactive curricula, and increased opportunities for nurses to practice [15–17]. Conversely, barriers to effective education and training may include a lack of time and financial support, as well as nurses' resistance to updating their knowledge and skills [18, 19].

The significant role of nursing care in stroke management is widely recognized, yet it has not received sufficient research attention. The results of one integrative review only confirmed the potential and feasibility of education/training for all stroke healthcare professionals, including doctors, neurologists, physiotherapists, paramedics, nurses, and dispatchers [20]. Nurses play a different role in stroke care than other healthcare professionals. Professional education/training can better address the professional development needs of nurses in these fields. At present, there has been no scope review on the education/training programs for nurses involved in stroke care. The impact of these education/training programs on nurses and the patients cared for by trained nurses has not been reported. Therefore, this review adopts a scoping review approach to summarize the existing scientific literature, examine the effects of stroke education and training on nurses involved in stroke care and their patients, and identify the factors that facilitate or hinder stroke education and training. Our findings may provide evidence for improving the quality of nurses caring for people with stroke.

## Materials and methods

This review employed a scoping review methodology [21] based on the Joanna Briggs Institute (JBI) method [22–24]. The Preferred Reporting Items for Systematic Reviews and Meta-Analyses extension for scoping reviews (PRISMA-ScR) checklist [25] and the 2020 PRISMA flow diagram were utilized. The following research questions were addressed: 1) What types of education/training programs have been developed for nurses involved in stroke patient care? 2) What are the effects of the education/training on nurses? 3) What are the effects of the education/training on patients? 4) What are the facilitators and barriers encountered in the implementation of the education/training?

### Search strategy

The research team developed a retrieval strategy based on the research purpose and content, and two members of the research team (ZYJ and MDF) independently conducted searches according to the retrieval strategy. The research team resolved the differences and ultimately determined the retrieval results. A comprehensive search was conducted across a range of databases, including PubMed, Cumulative Index to Nursing and Allied Health Literature, PsycINFO, Embase, Web of Science, Scopus, ProQuest Dissertations and Theses, Google Scholar, and Cochrane Library. The details of the search strategy, including those of the grey literature, are outlined in Additional file 1:  Appendix A. A hand-search of reference lists of all included studies was also performed to identify any additional articles that were not captured through database searches.

### Study eligibility

The JBI Participants Concept Context framework used to define the scoping review search strategy is described in Table 1.

**Table 1:** Search terms

Participants Concept Context Framework	Search terms selected
Participants	Nurse, Permanent nurse, Full-time nurse, Part-time nurse, Registered nurse, Registered practical nurse, Qualified and non-qualified rehab nurse, Nursing Staff, and Clinical Nurse Specialist
Concept	Educational Status, Education of Public Health Professionals, Education of Distance, Nursing Education Research, Vocational Education, Retraining, Professional Education, Inservice Training, Simulation Training, Teaching, Mentoring, Curriculum, and Learning
Context	Stroke, Cerebrovascular Accident, Apoplexy brain, and Vascular Accident brain

Inclusion criteria for this review included:

- Nurses working in the community or clinical settings with no age limitations;
- Study on education and training provided to nurses;
- Information on stroke patient care in various nursing settings;
- Reviews of the data, with no restrictions on information sources or study type. The study type included descriptive research, analytical research, trial research, thesis dissertations, and organizational reports;
- All studies on the impact of education/training on nurses involved in stroke care from publication to February 2023.
- Publications in English.

Exclusion criteria for this review included:

- The primary focus of education/training was not on nurses;
- Participants of nursing educators, nursing college instructors, nursing students, or graduate nurses;
- Reviews (e.g., integrative, systematic, and scoping reviews).

All retrieved records were imported into EndNote X9 to eliminate duplicate studies. Throughout the initial review and abstract screening, an iterative approach was utilized to further refine the inclusion/exclusion criteria to align the screening process with the focus of this review. We included peer-reviewed academic research articles that reported original data sources and employed diverse research methodologies. Grey literature resources included master's and doctoral dissertations found in ProQuest papers and degree databases, as well as reports obtained from relevant organizational websites, such as Google Scholar. The target participants comprised full-time nurses, part-time nurses, casual registered practical nurses, registered nurses, registered practical nurses, and permanent nurses. If the education/training participants primarily focused on nurses, it also encompassed healthcare assistants, nurse assistants, and other community health workers. Education/training reviews mainly involving nursing educators, nursing college instructors, nursing students, or graduate nurses working in medical environments (e.g., hospitals, clinical settings, nursing homes, and patient homes) and educational settings (e.g., nursing schools, colleges, and universities) were excluded. The articles on the impact of education/training on nurses other than nurses caring for stroke patients, as well as the impact of educated and trained nurses on the stroke patients they care for were excluded. Literature reviews (e.g., integrative, systematic, and scoping reviews) were excluded. Two members of the research team (XYZ and FSY) independently screen titles and abstracts from literature searches. The full texts of the articles with potentially relevant titles and abstracts were further screened by two independent researchers for articles that met the predefined inclusion criteria for the review. In case of disagreements or discrepancies between the two researchers regarding the inclusion of certain studies, they resolved them through discussion with the involvement of a third researcher (ZSZ).

### Data extraction

Based on previous literature and the research content of this review, the research team created a data extraction template, which included the author, year, country, review design, characteristics of staff participants (including nursing department, years of nursing practice, and completion of training/activities), characteristics of patient participants (including mean patient age, female/male ratio, and completion of training/activities), content of education and training programs, delivery format, delivery method, education and training providers, program frequency and duration, guiding theories, and main outcome measures. A pilot test was conducted in two papers. After confirming the efficacy of the template in extracting the required data for the review, the research team utilized it to extract data from all other relevant studies. The main consensuses of data extraction included the importance of ensuring data accuracy during extraction. Years of nursing practice refers to the overall duration of a nurse's participation in nursing work, rather than just focusing on stroke patient care. The number of staff participants, completion of training/activity, patient participants, mean patient age and patient sex must be strictly extracted according to the study's description. Data extraction included all essential data points to prevent loss and ensure the representation of complete information in this review. Didactic activities of education/training mainly involved lectures, brochures, demonstrations, self-directed e-learning, and videos. Interactive activities of education/training mainly involved simulation exercises, seminars, group discussions, and clinical practices. The main outcome measure was divided into two categories: nurse and quality of care. Nurse mainly refers to the impact of educational/training activities on nursing staff, encompassing their skills, job satisfaction, etc. Quality of care refers to the impact of education/training activities on patients, including their disease outcomes and satisfaction with care. Two researchers (HM and A·Y) independently extracted key data from eligible studies. If there are disagreements, they resolve them through discussion with the third researcher (SJ).

### Data analysis, synthesis, and charting of findings

The research characteristics and the scope and properties of available research are presented in the form of a numerical analysis table. To synthesize the research results, especially those related to the education/training of stroke nurses, as well as the role of stroke patients cared for by these educated and trained nurses, we identified a descriptive exploratory study published by Thompson et al. [26]. In their study, they identified the elements of education and training as follows: "Educates and supports staff to deliver safety, high-quality care, and make a valid contribution to ward patient safety," providing a useful framework for synthesizing these findings. Because of the focus of this review on exploring and participating in the education/training of nurses in the care of stroke patients, the research team also combined the three separate sections (i.e., essential requirements, knowledge and understanding, skills and ability) of the Stroke-Specific Education Framework [8]. Several crucial aspects and considerations of education and training in the nursing field were identified, encompassing the fundamental professional knowledge of nurses, nurses' clinical practice skills, impact on nurses, patient self-care abilities, care outcomes, patient satisfaction, barriers and facilitators to education and training, guiding the research team to synthesize findings on the impact of comprehensive education and training interventions on nurses participating in stroke care. The data was independently integrated by two researchers (ZXN and DYY). Any disagreements can be resolved through discussion or by a decision made by a third researcher (SDP).

As per the scoping review requirements, the quality assessment of the included articles is neither mandatory nor within the scope of the scoping review [24, 25]. Nevertheless, this paper adopted a critical perspective on the included literature due to the limitations of the overall paper evaluation approach.

## Results

### Search results

**Fig. 1:**
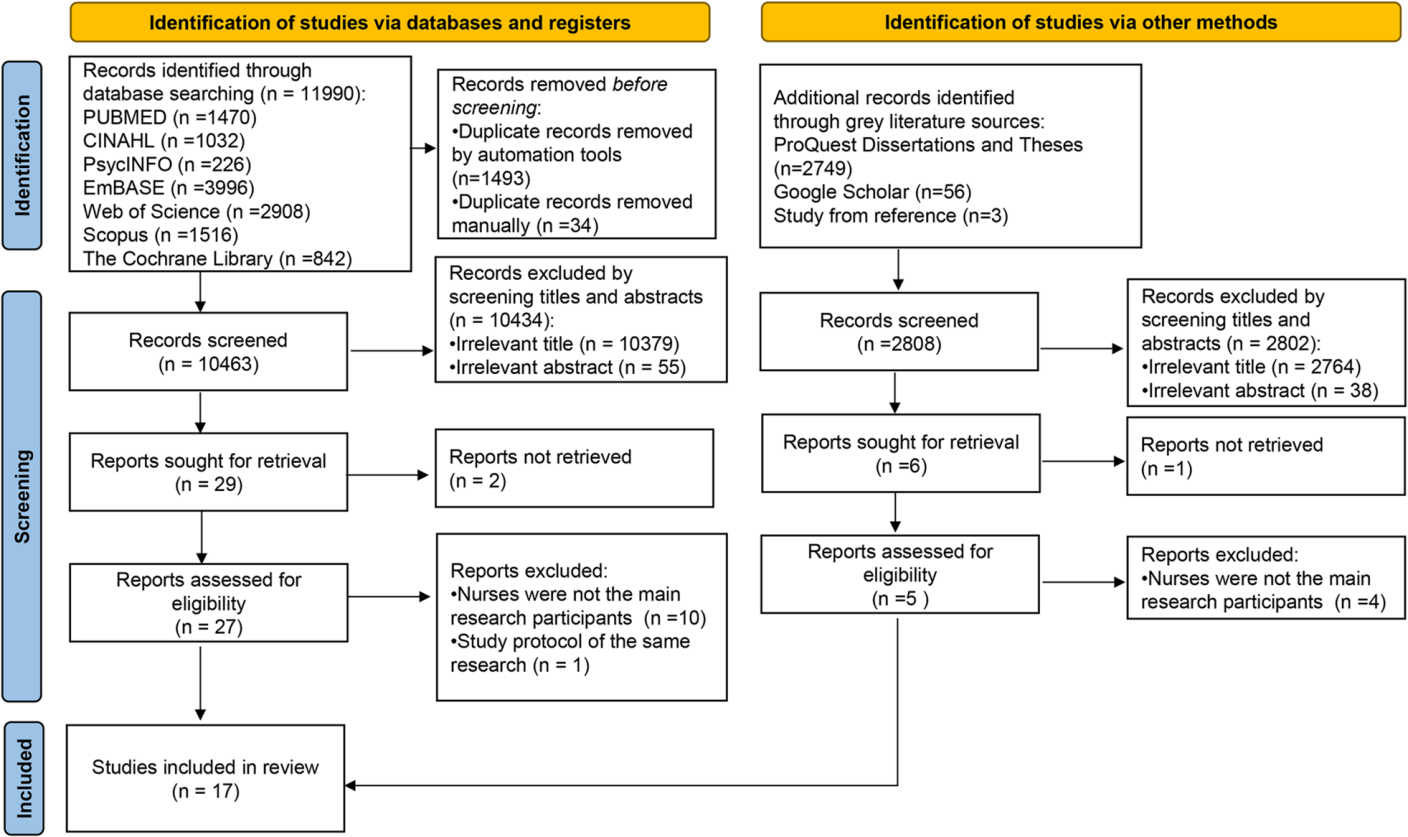
PRISMA flow chart illustrating the identification of literature for the scoping review

As shown in the PRISMA chart (Fig. 1), a total of 11,990 records were identified through databases and 2805 records were identified from ProQuest Dissertations and Theses, Google Scholar. After removing the duplicates (*n* = 1527), a total of 13,268 records were screened for the title and abstract, resulting in the exclusion of 13,239 records. The remaining 29 records and 3 additional full-text papers retrieved from reference lists were further assessed for eligibility. Ultimately, 17 records were included in this review.

### Basic characteristics of the studies

The records included in this review are presented in Table 2. Out of the 17 included studies, the majority (*n* = 14) were published in Western countries. Among these, five were from the United Kingdom [27–31] and four were from the United States [32–35]. The remaining studies were from Australia (*n* = 2) [36, 37], Switzerland (*n* = 2) [38, 39], Canada (*n* = 1) [40], and Asian countries with larger population bases and more developed healthcare systems, such as China (*n* = 1) [41], India (*n* = 1) [42], and Japan (*n* = 1) [43].

**Table 2:** Characteristics and programs of intervention for included studies

Author, Year; Country	study Type	Staff Participants	Nursing department	Years of nursing practice	Completion of training/activity	Patient Participants	Mean Patient Age (year)	Patients Female; Male (%)
Amato et al., 2006 USA [32]	Quasi-experimental pre-post measurement design	Nurses, N not stated	Nurses working in stroke rehabilitation units	NS	NS	NS	NS	NS
Booth et al., 2005 UK [28]	Quasi-experimental non-equivalent control group design	26 nurses	Nurses working in stroke rehabilitation units	NS	NS	37	NS	NS
Cadden, 2007 Australia [37]	Quasi-experimental pre-post measurement design	17 permanent nurses	Nurses working in stroke rehabilitation units	6 ~ 35 (SD = 14.27)	NS	NS	NS	NS
Chiu et al., 2009 China [41]	Quasi-experimental pre-post measurement design	129 nurses	Neurology specialty at neurology and neurosurgery wards	ICAI:79.3(SD ± 57.3) months; IVLP:79.3(SD ± 57.3) months	ICAI:48(64.7%); IVLP:44(65.6%)	NS	NS	NS
Chu et al., 2018 Canada [40]	Quasi-experimental pre-post measurement design	46 nurses including full-time, part-time, or casual RPNs or RNs	Nurses working in stroke rehabilitation units	11.4 (SD = 7.4)	46 (100%)	62	68	40; 60
Forster et al.,1999 UK [29]	Quasi-experimental pre-post measurement design	13 nurses	Nurses working in stroke rehabilitation units	NS	NS	Pre = 26 Post = 24	Pre = 78 Post = 77	Pre = 54;46 Post = 71; 29
Freeland et al., 2016 USA [34]	RCT	32 nurses	Medical Center	Control = 14.64 (SD = 11.96), Intervention = 16.53 (SD = 9.08)	*n* = 32,100% (2 weeks), *n* = 31,96.86% (4 weeks), and *n* = 29,90.63% ( 6 weeks)	2 (standardized patients)	NS	NS
Herr-Wilbert et al., 2010 Switzerland [38]	Cohort	16 nurses	The department of neurorehabilitation in the rehabilitation hospital	NS	NS	44	75	43; 57
Hisaka et al., 2021 Japan [43]	Cross-Sectional Web-Based Questionnaire Survey	1040 nurses	Stroke care unit	≤ 3(47.2%)	706 (81.70%)	NS	NS	NS
Jones et al., 1998 UK [27]	Quasi-experimental design	44 nurses and 15 HCAs	Two wards specialized in stroke rehabilitation and 4 were general medical wards	NS	59	38	73	74; 26
Jones et al., 2005 UK [30]	Cluster RCT	Nurses and HCAs, N not stated	Nurses working in stroke rehabilitation units	NS	NS	120	Control = 71, Intervention = 75	Control = 50;50, Intervention = 63; 37
Knippa et al., 2015 USA [35]	Quasi-experimental pre-post measurement design	223 nurses	neurology specialty of ICU	7.8(SD ± 7.07)	190 (85.20%)	N not stated (standardized patients)	NS	NS
Koka et al., 2020 Switzerland [39]	RCT	40 nurses	Emergency Medical Services	NS	39 (98%)	NS	NS	NS
Loft et al., 2018 USA [33]	Convergent, parallel, mixed-method design (purposively split into three groups)	36 nurses and nurse assistants	Nurses working in stroke rehabilitation units	< 2 (8); 2–5(4); > 5(19)	31 (86.11%)	NS	NS	NS
Middleton et al., 2011 Australia [36]	Cluster RCT	Nurses, N not stated	Acute Stroke Units (ASUs)	NS	NS	1696	< 65 Control = 28%, Intervention = 31%	40; 60
Roots et al., 2011 UK [31]	Knowledge introduction	Nurses, N not stated	Hyperacute stroke units	NS	NS	NS	NS	NS
Sylaja et al., 2021 India [42]	An open-label cluster RCT	Nurses and other community health workers, N not stated	Community Health Workers	NS	NS	Pre = 238 Post = 234	Control = 59.43 (SD = 11.07), Intervention = 59.77 (SD = 12.17)	Control = 28.3;71.7, Intervention = 30.7; 69.3

*HCAs* healthcare assistants, *RNs* registered nurses, *RPNs* registered practical nurses, *ICAI* interactive computer-assisted instruction, *IVLP* instructor-led videotape learning program, *ICU* Intensive Care Unit, *NS* No statement

Five studies utilized randomized trial designs, including two randomized controlled trials [34, 39] and three cluster randomized controlled trials [30, 36, 42]. One study followed a cohort study design [38], while another utilized a mixed-method design [33]. The remaining studies employed quasi-experimental designs: seven used pretest–posttest [27, 29, 32, 35, 37, 40, 41], one used a non-equivalent group [28], one conducted a Cross-Sectional Web-Based Questionnaire Survey [43], and one utilized knowledge introduction [31].

All studies included in this review considered nurses involved in stroke care as the primary participants for education/training. However, due to the necessity of program development, a small number of studies also included educated or trained healthcare assistants [27, 30], nurse assistants, and other community health workers [33].

Only five studies did not disclose the number of nurses involved in the education and training programs [30–32, 36, 42]. However, the remaining 12 studies provided information about the number of participating nurses. In total, approximately 1662 nurses took part in the education and training programs within the included studies The majority of these nurses were registered nurses or registered practical nurses (94.12%). Some studies also included permanent nurses [37], full-time nurses, part-time or casual nurses [40], health care assistants [27, 30], nurse assistants [33], and community health workers [42]. A few studies mentioned the number of years of nursing practice among the participating nurses, which varied from 2 to 35 years. Junior nurses represented the majority, accounting for 69.07% of the total. Furthermore, the completion rate of the education and training programs was consistently high for all nurses, ranging from 64.7% to 100%.

Out of the 17 studies included in this analysis, 15 implemented education and training interventions aimed at improving stroke patient care among nurses involved in stroke care. Eight of these studies specifically targeted nurses working in stroke rehabilitation [28–30, 32, 33, 37, 38, 40], while the remaining seven focused on nurses working in general wards [27], acute stroke units [36], medical centers [34], neurological ICUs [35], neurological wards [41], the community [42], and emergency medical services [39]. The other two studies had distinct focuses: one examined nurses' awareness and actual stroke care nursing practices through a web-based cross-sectional questionnaire in acute stroke units [43], and the other summarized knowledge gained from simulation training completed by nurses in hyperacute stroke units [31].

Nine studies reported information on selected patient characteristics [27–30, 34, 36, 38, 40, 42]. The known outcome analyses included a larger number of male than female patients (777 males and 563 females). One study did not impose an age restriction on stroke patients [36], while the remaining studies comprised patients aged between 59 and 78 years, who had experienced strokes and were admitted to diverse departments, including rehabilitation units, neurology units, stroke units, and various other departments.

The education/training programs focused on imparting the latest professional knowledge and addressing practical clinical care issues. To ensure accessibility to knowledge, the study developed the program. The education/training program commenced by providing an overview of stroke, covering its definition, etiology, influencing factors, treatment, general care, and rehabilitation. This was done to establish a foundational understanding of stroke-related nursing knowledge among the participating nurses [27–30]. Subsequently, specialized nursing knowledge on individuals with stroke was covered, including the administration of tissue-type plasminogen activator [35], cardiac monitoring [35, 37], and neurological assessment [31, 35, 39, 41]. Additionally, the education/training program was tailored to the specific characteristics of each nursing department to address practical clinical care concerns. Examples of such tailored training included management of blood glucose in stroke patients with diabetes mellitus [36]; rehabilitation of stroke patients with dysphagia [33–36]; rehabilitation of patients with urinary incontinence [38]; reducing physical restraints for stroke patients at risk of falls [32]; physiotherapy and secondary prevention [42]; stroke care; and interprofessional communication training for stroke unit doctors, dieticians, physiotherapists, and specialist nurses [31, 35, 40]. The relevance of these educational/training objectives was instrumental in their effectiveness.

Eight education/training methods were used across all the included studies, which were categorized into five formats: classroom lectures (face-to-face), book reading (learning manuals), simulation training (scenario-based and virtual simulations), demonstration teaching (presentations), and seminars (on-site, telephone/internet, and group/collective seminars). Most of the studies utilized multiple education/training methods, as there was no single method prevalent. Out of the included studies, eleven employed three or more types of education/training methods that positively influenced either the trained nurses or the quality of care [27–31, 33, 35–37, 40, 41]. Only two studies utilized a single education/training method, either face-to-face lectures [32] or demonstrations [34]. Additionally, one study primarily utilized online courses, providing flexibility for nurses to complete the education/training programs at their convenience [39]. The duration and frequency of the education/training programs were reported in these studies. Each education/training session ranged from 30 min to 2 h in length and from 1 working day to 15 months in duration. Most education/training programs were delivered multiple times. The education/training providers were predominantly clinical care specialists (*n* = 8) [27, 30, 32, 33, 35, 37, 40, 42], followed by physicians and occupational therapists (*n* = 6) [28, 29, 31, 34, 40, 41]. In addition, online education/training was provided by platform developers [39]. However, the providers in the remaining three studies were not specified [36, 38, 43].

Nine studies in this review examined education and training theories. Among them, four studies utilized rehabilitation guidelines, including the 2012 AHA Guidelines for Stroke Rehabilitation [35], the Australian National Clinical Guidelines for Stroke [36], and the International Consultation on Incontinence (ICI) guidelines [38], to enhance education and training in managing neurological incontinence in frail older individuals. Some of the studies used not only practice guidelines but also hospital fall prevention protocols to optimize the content of the education/training program [32]. Theoretical frameworks were used in three studies, such as the education/training program for nurses working in stroke rehabilitation units, where the content of the program applied the theory of complex interventions and a behavior change framework to enhance the practices of nurses [33]. Additionally, in the education/training of nurses working in stroke rehabilitation units, the content framework for the workshops was developed using the Aphasia Framework for Outcome Measurement and the person-centered education theory REAP (Relating well, Environmental manipulation. Ability-focused care and Personhood), to enhance experiential learning [40]. In addition, the education/training was based on social constructivism and cognitive constructivism concerning stroke disease. There was one treatment method category, which aims to guide the limb rehabilitation of people with stroke based on Bobath's method [28].

### Description of the impact of education/training measures on nurses

Adequately trained nurses have an improved ability to understand and apply their acquired knowledge and skills, resulting in more effective management and coordination of patient care, and ultimately enhancing their professional standing. The data are presented in Table 3.

**Table 3:** Summary of education and training interventions

Author, Year; Country	Content of Education and Training	Format of Delivery	Method of Delivery	Who Delivered Education and Training	Frequency and Duration	Guiding theory	Main Outcome Measure	
Amato et al., 2006 USA [32]	Restraint Reduction Program of administration, education, consultation, and feedback	Face-to-face lectures	NS	Clinical nurse specialist	Ongoing, duration not specified	Best evidence and practice guidelines from the hospital’s own Fall Prevention Protocol	2. Quality of care	The rates of physical restraint use and patient falls had decreased
Booth et al., 2005 UK [28]	Positioning, therapeutic handling of stroke patients, and facilitation of morning care activities	Formal lectures, simulated patient demonstrations, video demonstrations, and experiential learning	Didactic & Interactive	Senior physiotherapists and Occupational therapists	Two,3.5 h sessions (repeated)	Based on the Bobath (1990) approach to the treatment of stroke	1. Nurse 2. Quality of care	1. Improved the processes and practices of nurses during morning care activities; 2. Increased styles of interaction with nurses
Cadden, 2007 Australia [37]	Training of highly specialized cardiac monitors: cardiac anatomy and physiology, cardiac rhythm interpretation skills, and activities	Lectures, Imitation/practice/competition, workshop	Didactic & Interactive	Clinical nurse educator	2 months including 2 weeks of activities	Certain educational guidelines	1. Nurse 2. Quality of care	1. Skills, attitudes, and behaviors have been improved; 2. Increased safety of care
Chiu et al., 2009 China [41]	The use of the Chinese version of the National Institute of Health Stroke Scale (C-NIHSS)	Computer-based teaching and Video teaching, lecture, and demonstration	Didactic & Interactive	Bilingual and certified neurologist	ICAI:50 min; IVLP:70 min	NS	1. Nurse	Increased assessment skills and satisfaction
Chu et al., 2018 Canada [40]	Interprofessional (IP) Communication Training: common communication disorders, behavioral management	Pictures, demonstration, video clips, guided activities, role-play, workshop, Focus groups	Didactic & Interactive	1 Speech-language pathologist (SLP) and 1 academic nurse	Two workshops are eight months apart, the first being an eight-day workshop and the second being a half-day booster workshop	The Aphasia Framework, the REAP (Reading, Experiencing, Applying, Producing)model	Nurse	Nurses’ attitudes toward and knowledge about communication strategies improved, which enhanced their ability to care for stroke patients with communication disorders
Forster et al.,1999 UK [29]	A etiology of stroke, treatment philosophies, Positioning and transfers, factors influencing	Face-to-face lectures, videos, demonstrations, workshops/ group discussion	Didactic & Interactive	Physiotherapy lecturer and 3 senior physiotherapists	Multiple sessions, duration not specified (repeated)	NS	1. Nurse 2. Quality of care	1. Some improvements in clinical practice 2. There were no significant differences in patient outcomes
Freeland et al., 2016 USA [34]	Incidence of dysphagia, aspiration, stroke, distinction between screening and swallowing assessment, a review of screening items; hands-on simulated group practice session	Presentation, demonstration	Didactic & Interactive	Trainer of the medical simulation mannequin	Training at 2-week intervals, duration for 6 weeks	NS	Nurse	Over time, nursing skills have also improved
Herr-Wilbert et al., 2010 Switzerland [38]	Anatomy and physiology of the urinary tract, the urinary tract’s pathological conditions, and the various forms of urinary incontinence (UI), clinical skills to identify risks and signs of UI	Lecture, Manual/ workbook, practice	NS	NS	NS	The ICI Guidelines: Initial Management of Neurogenic Urinary Incontinence and Management of Urinary Incontinence in Frail Older Persons	2. Quality of care	Increasing the likelihood of positive results of rehabilitation of patients after cerebrovascular Accident
Hisaka et al., 2021 Japan [43]	NS	NS	NS	NS	NS	NS	NS	NS
Jones et al., 1998 UK [27]	The definition and etiology of stroke, factors influencing recovery, the multidisciplinary team’s role in rehabilitation, and the influence of ergonomics on movement and positioning	Face-to-face lectures, manual/ workbook, demonstrations	Didactic	Nursing lecturer	Two,2h lectures (repeated)	NS	1. Nurse 2. Quality of care	1. Improved knowledge and practice: perceiving the quality of the ward as a learning environment and their level of job satisfaction; 2. Improved the patients’ ability to adopt and maintain recommended positions
Jones et al., 2005 UK [30]	The definition and etiology of stroke, factors influencing recovery, the multidisciplinary team's role in rehabilitation, moving, handling, and positioning of patients	Practical workshops/ group discussion, face-to-face lectures, manual/ workbook	Didactic & Interactive	2 nursing lecturers	One, 1-day session plus two, 0.5-day sessions at five monthly intervals, 3 times	NS	1. Nurse 2. Quality of care	1. Nursing practice can be positively influenced through teaching; 2. Improvements in the quality of patient positioning, do not have any effect on patient outcomes
Knippa et al., 2015 USA [35]	tPA administration, vital sign monitoring, neurological assessments, dysphagia screening assessments, team communication	Lectures, virtual simulation, workshops, debriefing, role-play	Didactic & Interactive	A team of five ICU unit-based clinical nurse educators and a clinical nurse specialist	30-min scenario,30-min debriefing session,10-min orientation	2012 AHA Stroke Guidelines	Nurse	Simulation for experienced nurses can play a key role in improving patient care
Koka et al., 2020 Switzerland [39]	Enhance NIHSS knowledge acquisition	E-Learning and Video	Didactic & Interactive	4 platform developers	Four training sessions were organized on two different days	NS	Nurse	The use of an e-learning module shows promising results in teaching the NIHSS to paramedics
Loft et al., 2018 USA [33]	Nursing educational intervention for inpatient stroke rehabilitation	Face-to-face lectures, /internet /telephone workshops, Tasks, Training, video showing, Presentation, Role-play	Didactic & Interactive	Nurse specialist with at least a master’s degree	7‐week education programmer, three face‐to‐face workshops of 3 h duration with 2 weeks interval in between	The framework of the Medical Research Council (MRC) of the United Kingdom for developing complex interventions and the Behaviour Change Wheel	1. Nurse 2. Quality of care	1. 97% considered the educational program to be well-planned 2. A high level of satisfaction with the educational programmer in terms of its acceptability and feasibility
Middleton et al., 2011 Australia [36]	Clinical treatment protocols for the management of fever, hyperglycemia, and swallowing	Workshops/ discussions, face-to-face lectures, training CD, protocol, practical, on-the-job support, demonstrations	Didactic & Interactive	NS	Two sessions, duration not specified	Australia’s national clinical guidelines for stroke	2. Quality of care	Patients from intervention ASUs were significantly less likely to be dead or dependent (mRS ≥ 2) at 90 days than patients from control ASUs; and improved physical functioning; Concerning processes of care, Patients in intervention ASUs had a significantly lower mean temperature during the first 72 h of admission to the ASU compared with patients in the control ASUs
Roots et al., 2011 UK [31]	Technical skills (e. g. neurological examination and setup and delivery of thrombolysis), complex tasks (e. g. the transfer of patients while treatment is ongoing), and team training skills (e. g. communication skills and leadership)	Simulation training, a group debriefing, demonstration	Simulation & Interactive	Stroke specialist	4 simulated scenario (Each simulated scenario lasted up to 15 min), 1 group debriefing session (lasting approximately 40 min)	Both social and cognitive constructivism	Nurse	Improved non-technical skills: Communication, Leadership, Managing emergencies
Sylaja et al., 2021 India [42]	Management, secondary prevention of stroke; nursing care of the stroke survivors; physiotherapy aspects in care of stroke patients	NS	NS	The neurologist, nurse, and physiotherapist working in the comprehensive stroke center	45 min per session	NS	1. Nurse	Training improves the quality of health education provided by health services

*NS* No statement

A research team conducted education and training sessions to instruct nurses on the proper positioning of the unaffected limb to facilitate the recovery of limb function in stroke patients. The sessions began with a face-to-face lecture, addressing the definition and etiology of stroke, factors influencing recovery, the multidisciplinary team's role in rehabilitation, and the impact of ergonomics on movement and positioning. The nurses in both the intervention and non-intervention groups completed a questionnaire, revealing significantly higher knowledge scores among the nurses who received the intervention [27]. The education/training enhanced the nurses' fundamental professional knowledge. Furthermore, the nurses' knowledge has been expanded through various education and training programs. One such example is the restraint reduction program, aimed at preventing falls in stroke patients. The program equipped nurses with knowledge of risk factors for falls, policies regarding restraint and seclusion, and hospital philosophies on restraint use, and provided them with hands-on training on fall prevention [32].

In the education/training of nurses on clinical practice skills, the studies opted for more experiential methods rather than the traditional and monotonous training and assessment methods, aiming to enhance their clinical practice skills. For example, in the education/training on morning care activities [29], neurological assessment [39, 41], and cardiac monitoring [37] for patients with stroke, the studies used scenario simulation. This involved developing a series of nursing scenarios that closely resembled real-life situations, in which nurses were educated and trained to handle various nursing issues that may arise in a simulated work setting. This not only addressed the problem of the disconnect between theory and practice but also improved nurses' clinical practice adaptability and proficiency. Additionally, to identify and address any issues in the clinical practice of the educated/trained nurses, the studies also provided practical workshops in the education/training program, thereby bridging any gaps and enhancing the clinical practice skills of the nurses through repeated training [29, 30, 34, 36].

Furthermore, the interactive nature of the education/training program enabled nurses to refine their nonclinical skills, such as communication, leadership, and emergency management, through learning and practice [31, 40]. For example, in stroke rehabilitation, nurses who had received education/training were capable of employing suitable communication strategies to meet the needs of patients with communication difficulties, consequently mitigating their levels of frustration and agitation.

While the studies were highly specific in developing the education/training program, the involvement of the multidisciplinary team resulted in a detailed and well-thought-out education/training program. The education/training program resulted in the enhancement of the nurses' knowledge and skills, ultimately improving their self-confidence [27, 28, 37, 40]. As the training progressed, the nurses' attitudes and job satisfaction improved over time [27, 33, 37, 40, 41].

### Description of the impact of education/training measures on patients

Nine studies assessed the quality of care. Among these, seven studies demonstrated that education and training had a positive impact on improving the quality of care. It is important to note that the quality of care indicators evaluated in these studies varied significantly, leading to the categorization of assessment results into three main categories.

Patients with stroke cared for by nurses who had received a 3-month education/training can correctly position their limbs during daily self-care activities [27]. The Stroke Patient Restraint Reduction Program resulted in decreased use of physical restraints and lower fall rates among high-risk patients [32]. This positive outcome was achieved by providing education/training to nurses and closely monitoring their implementation of the care plan in their daily work.

The study results provided compelling evidence that better management of fever, hyperglycemia, and dysphagia in acute stroke patients during the first 72 h after admission had significant benefits for individuals with both mild and severe strokes [36, 38]. This improved management reduced mortality, improved physical function, and optimized the care process. Furthermore, integrating education/training with good ideas in an interactive and highly practical educational program was more likely to increase motivation and improve care outcomes. Additionally, integrating education/training with good concepts was more likely to motivate nurses to learn, thereby overcoming barriers to education/training and making programs more effective, thus improving care outcomes [37]. However, contradicting results were observed with a physiotherapist-led training program and a teaching intervention aimed at improving the posture of stroke patients. These interventions showed no effect on patient outcomes [29, 30].

The trained nurses not only improved their communication skills but also their handling of complex situations [33]. The trained nurses exhibited competence in prioritizing the physical and psychological needs of stroke patients, thus facilitating improved communication between patients and nurses [28]. As a result, this increased the acceptability of the nursing care plan and the overall satisfaction of patients with the nursing service [33].

### Description of the facilitators and barriers encountered in the implementation of education/training

As illustrated in Table 4, some of the education and training programs did not adequately consider the limited availability of nursing staff [38]. Additionally, they did not address the high volume of nursing tasks. For instance, in the consultation component of the education and training program aimed at reducing restraints and preventing falls in individuals with stroke, the clinical nurse specialist's check-ins with the nurses increased from once every two weeks to once a week. However, this increase in workload resulted in resistance from the trained nurses towards implementing the program. Resistance towards implementing the education and training program was observed [32]. A few nurses provided excuses for missing the education and training sessions, including illness, leave, and personal reasons [30, 36].

**Table 4:** Barriers and facilitators of education and training

Barriers	Country of published work [reference list ID]	Facilitators	Country of published work [reference list ID]
The feasibility of implementation is not considered		Professional needs	
• Limited human resources and numerous tasks • Staff resistance toward implementing a new program	[38] [30, 32, 36]	•Intrinsic drive to acquire knowledge •Desire for increased competence in clinical practice	[27, 28, 33, 34] [31]
Limited by cost-effectiveness		The support and participation of management	[29, 34, 36]
• Training cost is one of the important considerations • In the process of developing the course, more time and resources must be invested	[35] [41]	Education and training created an on-trolled environment	[31, 34]

Two studies examined the cost-effectiveness of developing education/training programs. Managers who thoroughly assess the costs of human and financial resources, as well as equipment, before developing an education/training program, often encounter barriers to implementation. These barriers may arise due to the cost–benefit imbalance or the inability to replicate the program in other areas [35, 41].

### Facilitators of education and training

The review identified key facilitators for implementing the education/training programs, including the support and participation of management, the need for specialized education and training, and the establishment of a controlled environment.

The support and involvement of managers were considered crucial in facilitating the implementation of education/training programs [34]. These education/training programs were achieved with limited staffing and without any additional resources and can be replicated in most district general hospitals [29, 36]. Replicating an education/training program can reduce work hours, better optimize care resources, reduce time wastage, and increase efficiency, thus facilitating hospital managers to actively support and explore stroke education/training programs.

Interviews identified a strong desire for specialized education/training among stroke nurses, while a questionnaire on their stroke knowledge revealed that all nurses obtained low scores [27, 28, 33, 34]. Furthermore, a subset of surveyed nurses emphasized the importance of providing education/training specifically tailored to the needs of stroke nurses to improve the efficiency of care. The lack of skills in stroke rehabilitation and the overwhelming of stroke nurses often result in limited nursing hours [31].

Simulation training is a paramount method utilized to train stroke nurses. Its purpose is to design a lifelike setting where nurses can hone their skills without jeopardizing patient safety. According to the nurses, certain practical exercises like assessing swallowing skills needed to be repeatedly practiced within a secure educational/training environment – a place that was benign for patients and free from the apprehension of repetition [31, 34]. Additionally, the nurses expressed a desire for instruction within a safe and regulated atmosphere, to acquire non-technical coping skills [31].

## Discussion

This review examines the impact of education/training on nurses involved in stroke care, as well as the barriers and facilitators in the education and training process. It specifically focuses on studies conducted in developing countries in both Western and Eastern regions. Education/training measures developed for nurses involved in stroke care were classified into five categories and yielded better educational outcomes when ≥ 3 categories of education/training methods were implemented. Management support and involvement played a key role in facilitating stroke education/training programs.

A key issue surrounding the implementation of stroke education/training programs is how to effectively translate the theoretical knowledge acquired by trained nurses into clinical practice. In this review, eleven studies employed three or more types of education/training methods that positively influenced either the trained nurses or the quality of care.With the increasing demand for education and training, and the advancements in technology, several different approaches have been developed. These methods cater to diverse groups of individuals with varying needs, resulting in different outcomes and costs. To date, face-to-face lectures remain the most popular method [44]. Despite being more traditional, they serve as the foundation for education and training programs [45, 46]. The advantages of face-to-face lectures include set learning times, systematic knowledge transfer, and opportunities for direct interaction [47]. The disadvantage of lecture-based education/training methods is that trainees are unable to actively participate and can only passively absorb information from the lecturer's presentation. This limitation hinders their thinking and absorption capabilities. Some researchers have suggested that while lecture-based methods can still be found in both face-to-face and online courses, they should only be used sparingly in hybrid courses [44]. Simulation training methods, on the other hand, offer a solution to these limitations. They involve more scenario-based teaching methods as opposed to traditional classroom-based methods, which stimulate trainees' interest in learning. Simulation training also allows trainees to gain perceptual knowledge and deepen their impressions. Moreover, it enables trainees to connect theoretical knowledge with practical knowledge, facilitating the development of deep and accurate concepts. Currently, scenario-based and virtual simulations are the most commonly utilized methods of simulation training in medical education/training [48–50]. While scenario-based simulations can be used when time and personnel constraints are not a concern [51, 52], the combination of simulation training and virtual simulations can improve the time and cost-effectiveness of education/training. However, the adoption of virtual simulation education/training platforms requires careful consideration of technological and platform-related issues [44, 53–55]. On the other hand, the discussion method focuses on developing trainees' problem-solving skills and analytical judgment, but it demands a high level of trainer experience and skill [54]. In the context of stroke education and training, a comprehensive approach, combining classroom lectures, simulation training, and discussion methods, is recommended to ensure a better translation of theoretical knowledge of stroke management into clinical practice in future education and training programs.

In addition, stroke survivors frequently experience sudden fluctuations in their condition, which makes their situation highly unpredictable. They require close medical monitoring and often suffer from physical dysfunction, cognitive impairment, and psychiatric issues. As a result, the content of stroke caregiver education/training programs needs to be highly specialized and complex [10–12]. To achieve optimal outcomes, it is essential to base stroke education/training on relevant theories. Currently, stroke education/training programs are undergoing development. Likely, a combination of theoretical models and guidelines for stroke care management will serve as the theoretical foundation for future stroke education/training. This approach aims to enhance the quality of stroke caregiver education and training.

This review provides further validation that education/training has a positive impact on the clinical practices of nurses. The findings indicate that well-trained nurses demonstrate improved abilities in recognizing and managing patients' conditions promptly, responding effectively to unexpected situations including cardiac arrest, respiratory failure, and shock [56], and experiencing a lower incidence of medical errors [57]. In addition, adequately trained nurses exhibit enhanced job satisfaction, and their patients report higher satisfaction levels with the nursing care they receive. This can be attributed to the increased capability of nurses to communicate effectively with patients and deliver personalized care tailored to their individual needs and preferences. Such personalized care fosters trust and support among patients, resulting in greater adherence to treatment plans and care management [58].

The primary objective of stroke education/training is to enhance the clinical outcomes of trained nurses, thereby improving the quality of care they provide to their patients. The findings of this review indicate that stroke education/training contributes to increased patient self-care, improved outcomes, and greater acceptance and satisfaction with the care program. These results not only underscore the feasibility and effectiveness of the existing stroke education/training models but also highlight the distinctive role of nurses in the management of stroke patients. However, only seven of the studies provided information on the patients attended to by the trained nurses, and there was a lack of standardized and comprehensive measures to evaluate the quality of care. These findings suggest that the current design of stroke education/training programs may have some limitations in assessing their effectiveness in clinical practice. In clinical research, it has become increasingly common to utilize measures of patients' attitudes toward their disease symptoms and their impact on their daily lives as a tool to evaluate outcomes and determine treatment and care options. Patient-reported outcomes are now recognized as the most direct measure of effectiveness in clinical practice. They provide a powerful reference for doctors in diagnosing and treating patients and are of great importance to clinical practice [59–61]. For future studies, it is recommended that researchers standardize the effectiveness of stroke education/training according to patient-reported outcomes.

This review highlights the significance of managerial support and involvement in promoting the implementation of stroke management education/training programs. One crucial aspect is the establishment of effective evaluation and monitoring mechanisms by governmental and healthcare institutions. These mechanisms ensure that the training programs and courses adhere to specific standards and quality requirements. Furthermore, they assess the knowledge and skills of professionals and initiate timely actions to address deficiencies and enhance overall standards [62]. Another critical point is that complex education/training necessitates efficient organizational management [63]. As stroke treatment technology advances and patient demand grows, education/training will continue to play a vital role. Therefore, governments and healthcare organizations should invest in improving the education/training of stroke professionals to deliver higher-quality care [64]. Simultaneously, professional and not-for-profit organizations should actively contribute by providing more training and educational resources for professionals, fostering continuous improvement in stroke treatment and care.

### Limitations

This is the first scoping review of the literature on the impact of education/training on nurses involved in stroke care. Nevertheless, this review does have certain limitations. Firstly, we solely included literature published in English, possibly excluding significant studies, thereby constraining the scope of the findings. Secondly, a few studies failed to provide information on the number, age, and years of experience of the participating nurses, which somewhat hinders the generalizability of the results. Lastly, some studies encompassed in this review were over a decade old, rendering the results less contemporaneous.

## Conclusions

This review comprehensively examines the potential impact of education and training on nurses caring for stroke patients, the challenges they may face, and the key success factors. The results of the study reveal several exciting findings. Firstly, systematic education and continuous training undeniably enhance nurses' knowledge and clinical skills in stroke care. Well-trained nurses not only gain deeper insights and apply their knowledge and skills, but also translate these abilities into tangible improvements in patient care and care processes, thereby enhancing their professional development. Secondly, the effectiveness of education and training boosts nurses' confidence and job satisfaction. This enables them to empathize with patients and provide personalized care addressing their physical and emotional needs. Subsequently, this leads to a significant increase in patients' recognition and overall satisfaction with nursing services. Additionally, management support and involvement are crucial for the success of the education and training program. Simultaneously, the establishment of a comprehensive evaluation and monitoring system is crucial for maintaining high standards and quality in nursing education and training. Despite the positive impact of current education and training mechanisms on nurses' professional practice, there are still limitations in evaluating their effectiveness in clinical practice. Hence, future research should focus on developing more objective, standardized, and comprehensive evaluation strategies to thoroughly assess the effectiveness of education and training programs.

### Supplementary Information

**Additional file 1. **Appendix A provides the full list of search terms.

## Data Availability

No underlying data was collected or produced in this review.

## Abbreviations

PRISMA: Preferred Reporting Items for Systematic reviews and Meta-Analyses
HCAs: Healthcare assistants
RNs: Registered nurses
RPNs: Registered practical nurses
ICAI: Interactive computer-assisted instruction
IVLP: Instructor-led videotape learning program
ICU: Intensive Care Unit
NS: No statement
ICI: International Consultation on Incontinence
REAP: Relating well, Environmental manipulation, Ability-focused care, and Personhood
PROs: Patient-reported outcomes
