# Supplementary material for: The impact of education/training on nurses caring for patients with stroke: a scoping review
Source: BMC Nurs. 2024 Feb 2;23:90. doi: 10.1186/s12912-024-01754-x (PMC10835862; doi:10.1186/s12912-024-01754-x)
Supplement: Supplementary file 1 — Additional file 1. Appendix A provides the full list of search terms. [file 12912_2024_1754_MOESM1_ESM.docx]

| **Appendix A Literature search strategy** | | |
| --- | --- | --- |
| Database (No time limit) | Search terms | Number of articles |
| Pubmed | ("nurse s"[All Fields] OR "nurses"[MeSH Terms] OR "nurses"[All Fields] OR "nurse"[All Fields] OR "nurses s"[All Fields]) AND ("stroke"[MeSH Terms] OR "stroke"[All Fields] OR "strokes"[All Fields] OR "stroke s"[All Fields] OR ("stroke"[MeSH Terms] OR "stroke"[All Fields] OR ("cerebrovascular"[All Fields] AND "accident"[All Fields]) OR "cerebrovascular accident"[All Fields]) OR (("apoplexies"[All Fields] OR "stroke"[MeSH Terms] OR "stroke"[All Fields] OR "apoplexy"[All Fields]) AND ("brain"[MeSH Terms] OR "brain"[All Fields] OR "brains"[All Fields] OR "brain s"[All Fields])) OR ("stroke"[MeSH Terms] OR "stroke"[All Fields] OR ("vascular"[All Fields] AND "accident"[All Fields] AND "brain"[All Fields]) OR "vascular accident brain"[All Fields]) OR ("stroke"[MeSH Terms] OR "stroke"[All Fields] OR "cva"[All Fields])) AND ("educability"[All Fields] OR "educable"[All Fields] OR "educates"[All Fields] OR "education"[MeSH Subheading] OR "education"[All Fields] OR "educational status"[MeSH Terms] OR ("educational"[All Fields] AND "status"[All Fields]) OR "educational status"[All Fields] OR "education"[MeSH Terms] OR "education s"[All Fields] OR "educational"[All Fields] OR "educative"[All Fields] OR "educator"[All Fields] OR "educator s"[All Fields] OR "educators"[All Fields] OR "teaching"[MeSH Terms] OR "teaching"[All Fields] OR "educate"[All Fields] OR "educated"[All Fields] OR "educating"[All Fields] OR "educations"[All Fields] OR ("education"[MeSH Subheading] OR "education"[All Fields] OR "training"[All Fields] OR "education"[MeSH Terms] OR "train"[All Fields] OR "train s"[All Fields] OR "trained"[All Fields] OR "training s"[All Fields] OR "trainings"[All Fields] OR "trains"[All Fields]) OR ("education"[MeSH Subheading] OR "education"[All Fields] OR "teaching"[All Fields] OR "teaching"[MeSH Terms] OR "teaches"[All Fields] OR "teach"[All Fields] OR "teachings"[All Fields] OR "teaching s"[All Fields]) OR ("mentor s"[All Fields] OR "mentored"[All Fields] OR "mentoring"[MeSH Terms] OR "mentoring"[All Fields] OR "mentors"[MeSH Terms] OR "mentors"[All Fields] OR "mentor"[All Fields]) OR ("curriculum"[MeSH Terms] OR "curriculum"[All Fields] OR "curricula"[All Fields] OR "curriculums"[All Fields] OR "curriculum s"[All Fields] OR "education"[MeSH Subheading] OR "education"[All Fields]) OR ("learning"[MeSH Terms] OR "learning"[All Fields] OR "learn"[All Fields] OR "learned"[All Fields] OR "learning s"[All Fields] OR "learnings"[All Fields] OR "learns"[All Fields])) | 1470 |
| Cochrane | #1 MeSH descriptor: [nurses] explode all trees #2 (nurse):ti,ab,kw  #3 #1 OR #2  #4 MeSH descriptor: [Education] explode all trees  #5 MeSH descriptor: [Teaching] explode all trees  #6 MeSH descriptor: [Mentoring] explode all trees  #7 MeSH descriptor: [Curriculum] explode all trees  #8 MeSH descriptor: [Learning] explode all trees  #9 #4 OR #5 OR #6 OR #7 OR #8 OR  #10 (Education):ti,ab,kw OR (Training):ti,ab,kw OR (Teaching):ti,ab,kw OR (Mentoring):ti,ab,kw OR (Curriculum):ti,ab,kw OR (Learning):ti,ab,kw (Word variations have been searched)  #11 #9 OR #10  #12 MeSH descriptor: [Stroke] explode all trees  #13 (Stroke):ti,ab,kw OR (Cerebrovascular Accident):ti,ab,kw OR (Apoplexy brain):ti,ab,kw OR (Vascular Accident brain):ti,ab,kw OR (CVA):ti,ab,kw(Word variations have been searched)  #14 #12 OR #13  #15 #3 AND #11 AND #14 | 842 |
| PsycINFO | S1 AB nurs*  S2 SU "nurses"  S3 S1 OR S2  S4 AB Educat* or Training* or Teaching* or Mentoring* or Curriculum* or Learning*  S5 SU Education OR SU Teaching OR SU Curriculum OR SU Learning OR SU Mentoring OR SU Training  S6 S4 OR S5  S7 AB Stroke* or "Cerebrovascular Accident" or "Apoplexy brain" or "Vascular Accident brain" or "CVA"  S8 SU "Stroke"  S9 S7 OR S8  S10 S3 AND S6 AND S9 | 226 |
| Web of Science | TS=(Nurs*) AND (((((TS=(Educa*)) OR TS=(Train*)) OR TS=(Teach*)) OR TS=(Mentor*)) OR TS=(curriculum)) OR TS=(Learn*) AND ((((TS=(Stroke)) OR TS=(Cerebrovascular Acciden)) OR TS=(Apoplexy brain)) OR TS=(Vascular Accident brain)) OR TS=(CVA) | 2908 |
| EMBASE | #1 'nurse'/exp OR 'nurse'  #2 'nurs*':ab,ti,kw  #3'education'/exp OR 'education' OR 'training'/exp OR 'training' OR 'teaching'/exp OR 'teaching' OR 'mentoring'/exp OR 'mentoring' OR 'curriculum'/exp OR 'curriculum' OR 'learning'/exp OR 'learning'  #4 'education':ab,ti,kw OR 'training':ab,ti,kw OR 'teaching':ab,ti,kw OR 'mentoring':ab,ti,kw OR 'curriculum':ab,ti,kw OR 'learning':ab,ti,kw  #5 'cerebrovascular accident'/exp OR 'cerebrovascular accident'  #6 'stroke':ab,ti,kw OR 'cerebrovascular accident':ab,ti,kw OR 'apoplexy brain':ab,ti,kw OR 'vascular accident brain':ab,ti,kw OR 'cva':ab,ti,kw  #7 #1 OR #2  #8 #3 OR #4  #9 #5 OR #6  #10 #7 AND #8 AND #9 | 3996 |
| CINAHL | S1 AB nurse or nurses or nursing  S2 MH "nurses"  S3 S1 OR S2  S4 AB Educat* or Train* or Teach* or Mentor* or Curriculum* or Learn*  S5 (MH "Education") OR (MH "Teaching") OR (MH "Curriculum") OR (MH "Learning")  S6 S4 OR S5  S7 AB Stroke* or "Cerebrovascular Accident" or "Apoplexy brain" or "Vascular Accident brain" or "CVA"  S8 MH "Stroke"  S9 S7 OR S8  S10 S3 AND S6 AND S9 | 1032 |
| Scopus | ( TITLE-ABS-KEY ( nurse* ) ) AND ( ( TITLE-ABS-KEY ( educat* ) OR TITLE-ABS-KEY ( train* ) OR TITLE-ABS-KEY ( teach* ) OR TITLE-ABS-KEY ( mentor* ) OR TITLE-ABS-KEY ( curriculum* ) OR TITLE-ABS-KEY ( learn* ) ) ) AND ( ( TITLE-ABS-KEY ( stroke* ) OR TITLE-ABS-KEY ( "Cerebrovascular Accident" ) OR TITLE-ABS-KEY ( "Apoplexy brain" ) OR TITLE-ABS-KEY ( "Vascular Accident brain" ) OR TITLE-ABS-KEY ( "CVA" ) ) ) | 1516 |
| ProQuest Dissertations and Theses | (summary(nurse*) OR mainsubject(nurse)) AND ((summary(Educat*) OR summary(Train*) OR summary(Teach*) OR summary(Mentor*) OR summary(Curriculum*) OR summary(Learn* )) OR (mainsubject(Education) OR mainsubject(Training) OR mainsubject(Teaching) OR mainsubject(Mentoring) OR mainsubject(Curriculum) OR mainsubject(Learning))) AND ((summary(Stroke*) OR summary("Cerebrovascular Accident") OR summary("Apoplexy brain") OR summary("Vascular Accident brain") OR summary("CVA")) OR (mainsubject(Stroke) OR mainsubject("Cerebrovascular Accident") OR mainsubject("Apoplexy brain") OR mainsubject("Vascular Accident brain") OR mainsubject("CVA"))) | 2749 |
| Google Scholar | allintitle: nurse AND (Education OR Training OR Teaching OR Mentoring OR Curriculum OR Learning) AND "Stroke" | 56 |
